# High serum uric acid within the normal range is a useful predictor of hypertension among Japanese community-dwelling elderly women

**DOI:** 10.1186/s40885-020-00155-x

**Published:** 2020-10-15

**Authors:** Ryuichi Kawamoto, Daisuke Ninomiya, Taichi Akase, Kikuchi Asuka, Teru Kumagi

**Affiliations:** 1 Department of Community Medicine, Ehime University Graduate School of Medicine, Shitsukawa, Toon-city, Japan; 2 Department of Internal Medicine, Seiyo Municipal Nomura Hospital, 9-53 Nomura, Nomura-cho, Seiyo-city, Japan

**Keywords:** Serum uric acid, Hypertension, Risk factor, Women, Community-dwelling person

## Abstract

**Background:**

The risk associated with serum uric acid (SUA) levels when within the normal range is unknown. This study aims to examine whether SUA within the normal range is a predictor of hypertension.

**Methods:**

The subjects comprised 704 men aged 71 ± 9 (mean ± standard deviation) years and 946 women aged 70 ± 8 years recruited for a survey at the community based annual medical check-up. The main outcome was the presence of hypertension (antihypertensive medication and/or having SBP ≥140 mmHg and/or DBP ≥90 mmHg).

**Results:**

At baseline, 467 (66.3%) men and 608 (64.3%) women had hypertension. Comparing to lowest quartile in women (SUA-1, uric acid < 4.1 mg/dL), the unadjusted odds ratios (ORs) [95% confidence interval (CI)] for hypertension of SUA-2 (4.1 to 4.7 mg/dL), SUA-3 (4.8 to 5.4 mg/dL), and SUA-4 (≥5.5 mg/dL) were 1.11 (0.78–1.59), 1.75 (1.20–2.55), and 1.89 (1.30–2.77), respectively. These associations were apparent even after adjustments for age, but ORs were attenuated after adjusting for all confounding factors. During a follow-up of 3.0 years, there were 35 (24.0%) hypertension cases in men and 51 (20.8%) in women. In women only, a significant association between increased SUA categories and incidence of hypertension was observed, and the multivariate-ORs (95% (CI) for incident hypertension of SUA-3 (4.5–5.2 mg/dL) and SUA-4 (≥5.3 mg/dL) were 2.23 (0.81–6.11) and 3.84 (1.36–10.8), respectively.

**Conclusions:**

These results suggest that baseline SUA within the normal range could be an important predictor for incidence of hypertension in Japanese community-dwelling elderly women.

## Background

Hypertension has increased significantly with time, and the increasing prevalence is an important public health concern in Japan [1] and other countries [2–4] because of the high prevalence and strong association with cardiovascular disease (CVD). However, approximately 90% of hypertensive cases are essential hypertension, the etiology of its onset is not fully understood.

Although the etiology of essential hypertension is unknown, serum uric acid (SUA), the final product of the purine metabolism, has been hypothesized to activate intrarenal renin-angiotensin system (RAS), which can cause injury to pre-renal blood vessels [5]. For decades elevated SUA levels were mainly considered a result rather than a cause of renal dysfunction [6]. However lots of experimental and epidemiological studies have shown that high SUA in humans is associated with systemic inflammation [7], and hypertension [8–11]. These studies provide direct evidence that SUA may be a true mediator of hypertension and its progression. Some studies have shown that blood pressure (BP) is lowered by SUA lowering medications (e.g., allopurinol or probenecid) [12, 13]. However, as the association between SUA level and incident hypertension is affected by race, gender, age [14], body mass index (BMI) [15], lipids [16], and other confounding factors, there are some studies showing conflicting results [17]. In addition, whether targeting treatment based on SUA levels might affect clinical outcomes is still being studied [18] and the risk associated with SUA levels within the normal range is unknown. We evaluated the relationship between baseline SUA within the normal range and potential risk factors such as hypertension using cross-sectional and prospective cohort data from community-dwelling persons.

## Methods

### Study participants and data collection

The present study was a prospective cohort designed as part of the Nomura study [19]. The study population was recruited through a community-based annual survey process from the Nomura Health and Welfare Center in a rural town in Ehime prefecture, Japan. This study was started in 2014, and included 1832 community-dwelling participants aged 22–95 years. Follow-up assessment cycles are being performed every 3 years.

**Fig. 1:**
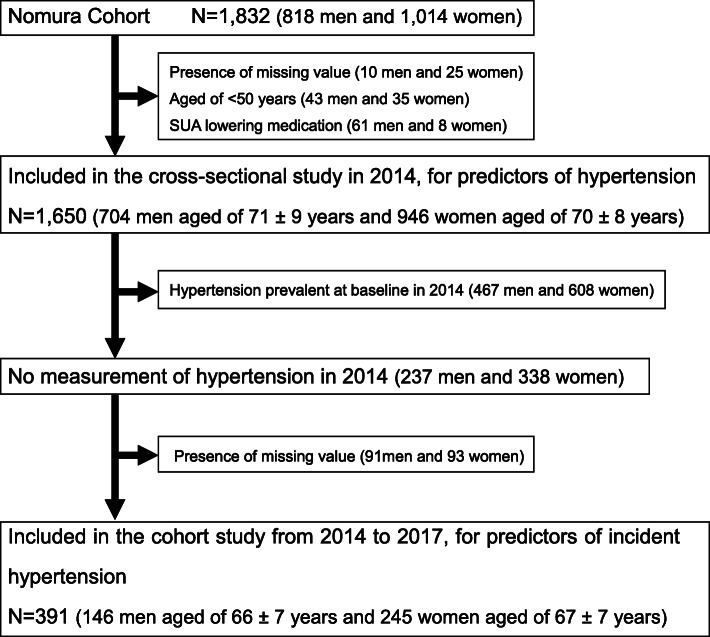
Flowchart. For the cross-sectional analyses, data of the 2014 cycle (*n* = 1650) were used as hypertension was measured in this cycle. For the longitudinal analyses, a sub-cohort of the 2014 cycle was used including only participants in whom hypertension was not prevalent at baseline in 2014 (*n* = 391)

Blood samples were obtained only from the respondents who participated in the medical interview at baseline. Information on medical history, current condition, and medication (such as antihypertensive, antilipidemic, antidiabetic, and SUA lowering medications) was obtained by interview using structured questionnaires. We excluded participants with a missing value (10 men and 25 women), aged < 50 years (men, *N* = 43; women, *N* = 35) and on an SUA lowering medication (men, *N* = 61; women, *N* = 8). For the cross-sectional analyses, data of the 2014 (*n* = 1650) were used as hypertension was measured in this cycle. For the longitudinal analyses, a sub-cohort of the 2014 cycle was used including only participants in whom hypertension was absent at baseline in 2014 (*n* = 391). Figure 1 shows a flowchart of the inclusion of participants. This study complies with the Declaration of Helsinki and written informed consent was obtained from each subject with the approval of Ehime University Medical School Ethics Committee. (Institutional Review Board: 1402009).

### Evaluation of risk factors

Information on demographic characteristics and risk factors was collected using the clinical files at baseline and the 3-year follow-up. BMI was calculated by dividing body weight (kilogram) by the square of height (meters). Smoking status was defined as the number of cigarette packs per day multiplied by the number of years smoked (pack-year), and the subjects were classified into never smokers, past smokers, light smokers (< 20 pack-year) and heavy smokers (≥20 pack-year). Daily Alcohol consumption was measured using a sake brewing unit with 1 unit being equivalent to 22.9 g of ethanol, and the subjects were classified into never drinkers, occasional drinkers (< 1 unit/day), daily light drinkers (1–2 units/day), and daily heavy drinkers (2–3 units/day). We measured systolic BP (SBP) and diastolic BP (DBP) of the upper right arm of the subjects in the sedentary position using an automatic oscillometric BP recorder while sitting after having rested for at least 5 min. Appropriate cuff bladder size was determined at each visit based on arm circumference. The mean of two consecutive measurements was used for the analysis. Normotension was defined as not being on any antihypertensive medication and having a SBP < 120 mmHg and DBP < 80 mmHg. Prehypertension was defined as not being on any antihypertensive medication and having a SBP of 120 to 139 mmHg and/or DBP 80 to 89 mmHg. Hypertension was defined as being on antihypertensive medication and/or having SBP ≥140 mmHg and/or DBP ≥90 mmHg according to the definitions of the Joint National Committee 7 (JNC7). Triglycerides (TG), high-density lipoprotein cholesterol (HDL-C), low-density lipoprotein cholesterol (LDL-C), SUA, hemoglobin A1c (HbA1c), and creatinine (Cr) were measured while fasted. Estimated glomerular filtration ratio (eGFR) was calculated using Chronic Kidney Disease Epidemiology Collaboration (CKD-EPI) equations modified by a Japanese coefficient: Male, Cr ≤0.9 mg/dl, 141 × (Cr/0.9) ^–0.411^ × 0.993 ^age^ × 0.813; Cr > 0.9 mg/dl, 141 × (Cr/0.9) ^–1.209^ × 0.993 ^age^ × 0.813; Female, Cr ≤0.7 mg/dl, 144 × (Cr/0.7) ^–0.329^ × 0.993 ^age^ × 0.813; Cr > 0.7 mg/dl, 144 × (Cr/0.7) ^–1.209^ × 0.993 ^age^ × 0.813 [20]. Moreover, ischemic stroke, ischemic heart disease, and peripheral vascular disease were defined as cardiovascular diseases (CVD).

### Statistical analysis

All values are expressed as mean ± standard deviation (SD), unless otherwise specified, and for parameters with non-normal distribution (such as TG, HbA1c), the data is given as the median (quartile range) value. For all analyses, parameters with non-normal distribution were used after logarithmic transformation. The subjects were divided into four groups based on quartiles of baseline SUA in each cross-sectional (men/women: SUA-1, < 5.2/< 4.1; SUA-2; 5.2–5.9/4.1–4.7; SUA-3, 6.0–6.7/4.8–5.4, SUA-4, ≥6.8/≥5.5 mg/dL) and cohort study (SUA-1, < 5.3/< 4.0; SUA-2; 5.3–5.8/4.0–4.4; SUA-3, 5.9–6.4/4.5–5.2, SUA-4, ≥6.5/≥5.3 mg/dL). Statistical analysis was performed using IBM SPSS Statistical Version 26 (Statistical Package of Social Science Japan, Tokyo, Japan). Differences in means and prevalence among baseline findings were analyzed by Student’s t-test or ANOVA for continuous data, and χ^2^ test for categorical data. Multiple logistic regression analysis was used to evaluate the contribution of the baseline SUA categories and confounding factors (i.e., gender, age, smoking habits, alcohol consumption, and prevalence of CVD, LDL-C, TG, HDL-C, antilipidemic medication, HbA1c, antidiabetic medication, and eGFR) for prevalence of hypertension in the cross-sectional study and incidence of hypertension in the cohort study. A value of *p* < 0.05 was considered significant.

## Results

### Baseline characteristics of subjects by gender

Baseline characteristics of the subjects are illustrated in Table 1. The subjects comprised 704 men aged 71 ± 9 years and 946 women aged 70 ± 8 years. BMI, smoking habit, alcohol consumption, history of CVD, DBP, TG, HbA1c, presence of antidiabetic medication, and SUA were significantly higher in men than in women, but HDL-C, LDL-C, presence of antilipidemic medication, and eGFR were higher in women than in men. There was no inter-group difference regarding age, SBP, and presence of antihypertensive medications. As shown in Table 2, in the cohort study, baseline BMI, smoking habit, alcohol consumption, DBP, TG, presence of antidiabetic medication, and SUA were significantly higher in men than in women, but HDL-C, LDL-C, and presence of antilipidemic medication were higher in women than in men.

**Table 1:** Baseline characteristics of study subjects in the cross-sectional study

Baseline Characteristics ***N*** = 1650	Men ***N*** = 704	Women ***N*** = 946	***P***-value*
Age (years)	71 ± 9	70 ± 8	0.578
Body mass index (kg/m^2^)	23.1 ± 3.0	22.6 ± 3.2	**< 0.001**
Smoking habit (never/past/light/heavy (%))	14.3/4.1/38.8/42.8	0.4/0.7/2.0/96.8	**< 0.001**
Alcohol consumption (never/occasional/light/heavy (%))	34.7/16.8/22.9/25.7	2.0/4.4/21.9/71.7	**< 0.001**
History of Cardiovascular disease (%)	10.2	4.2	**< 0.001**
Systolic blood pressure (mmHg)	136 ± 17	137 ± 18	0.517
Diastolic blood pressure (mmHg)	79 ± 10	77 ± 10	**< 0.001**
Antihypertensive medication (%)	44.9	44.2	0.802
Triglycerides (mg/dl)	89 (67–130)	87 (65–116)	**< 0.001**
HDL cholesterol (mg/dl)	62 ± 16	69 ± 17	**< 0.001**
LDL cholesterol (mg/dl)	114 ± 29	125 ± 29	**< 0.001**
Antilipidemic medication (%)	13.2	29.5	**< 0.001**
Hemoglobin A 1c (%)	5.7 (5.4–6.0)	5.7 (5.5–5.9)	**0.041**
Antidiabetic medication (%)	13.8	5.5	**< 0.001**
Estimated GFR (ml/min/1.73 m^2^/year)	70.0 ± 12.2	72.2 ± 10.6	**< 0.001**
Serum uric acid (mg/dL)	5.9 ± 1.3	4.7 ± 1.1	**< 0.001**

*HDL* High-density lipoprotein, *LDL* Low-density lipoprotein, *GFR* Glomerular filtration ratio. Data presented are mean ± standard deviation. Data for triglycerides and HemoglobinA1c is skewed, and presented as median (interquartile range) values. * *P*-value: Student’s t-test for the continuous variables or the χ^2^ -test for the categorical variables. Bold values indicate significance (*p* < 0.05)

**Table 2:** Baseline characteristics of study subjects in the cohort study

Baseline Characteristics ***N*** = 391	Men ***N*** = 146	Women ***N*** = 245	***P***-value*
Age (years)	66 ± 7	67 ± 7	0.287
Body mass index (kg/m^2^)	22.1 ± 2.3	21.3 ± 2.8	**0.005**
Smoking habit (never/past/light/heavy (%))	39.7/34.2/5.5/20.5	96.7/1.2/0.8/1.2	**< 0.001**
Alcohol consumption (never/occasional/light/heavy (%))	25.3/26.0/13.7/34.9	67.8/24.9/4.5/2.9	**< 0.001**
History of Cardiovascular disease (%)	3.4	2.4	0.548
Systolic blood pressure (mmHg)	121 ± 11	122 ± 12	0.608
Diastolic blood pressure (mmHg)	74 ± 8	71 ± 8	**< 0.001**
Antihypertensive medication (%)	0	0	1.000
Triglycerides (mg/dl)	85 (63–131)	79 (59–107)	**0.007**
HDL cholesterol (mg/dl)	63 ± 17	71 ± 18	**< 0.001**
LDL cholesterol (mg/dl)	116 ± 29	127 ± 28	**< 0.001**
Antilipidemic medication (%)	8.2	22.0	**< 0.001**
Hemoglobin A 1c (%)	5.6 (5.4–5.9)	5.6 (5.4–5.8)	0.566
Antidiabetic medication (%)	9.6	1.6	**0.001**
Estimated GFR (ml/min/1.73 m^2^/year)	74.6 ± 8.9	75.4 ± 9.3	0.397
Serum uric acid (mg/dL)	5.9 ± 1.2	4.5 ± 1.0	**< 0.001**

*HDL* High-density lipoprotein, *LDL* Low-density lipoprotein, *GFR* Glomerular filtration ratio. Data presented are mean ± standard deviation. Data for triglycerides and HemoglobinA1c is skewed, and presented as median (interquartile range) values. * *P*-value: Student’s t-test for the continuous variables or the χ^2^ -test for the categorical variables. Bold values indicate significance (*p* < 0.05)

### Prevalence of prehypertension and hypertension in subjects according to baseline SUA categories by gender in the cross-sectional and cohort studies

**Fig. 2:**
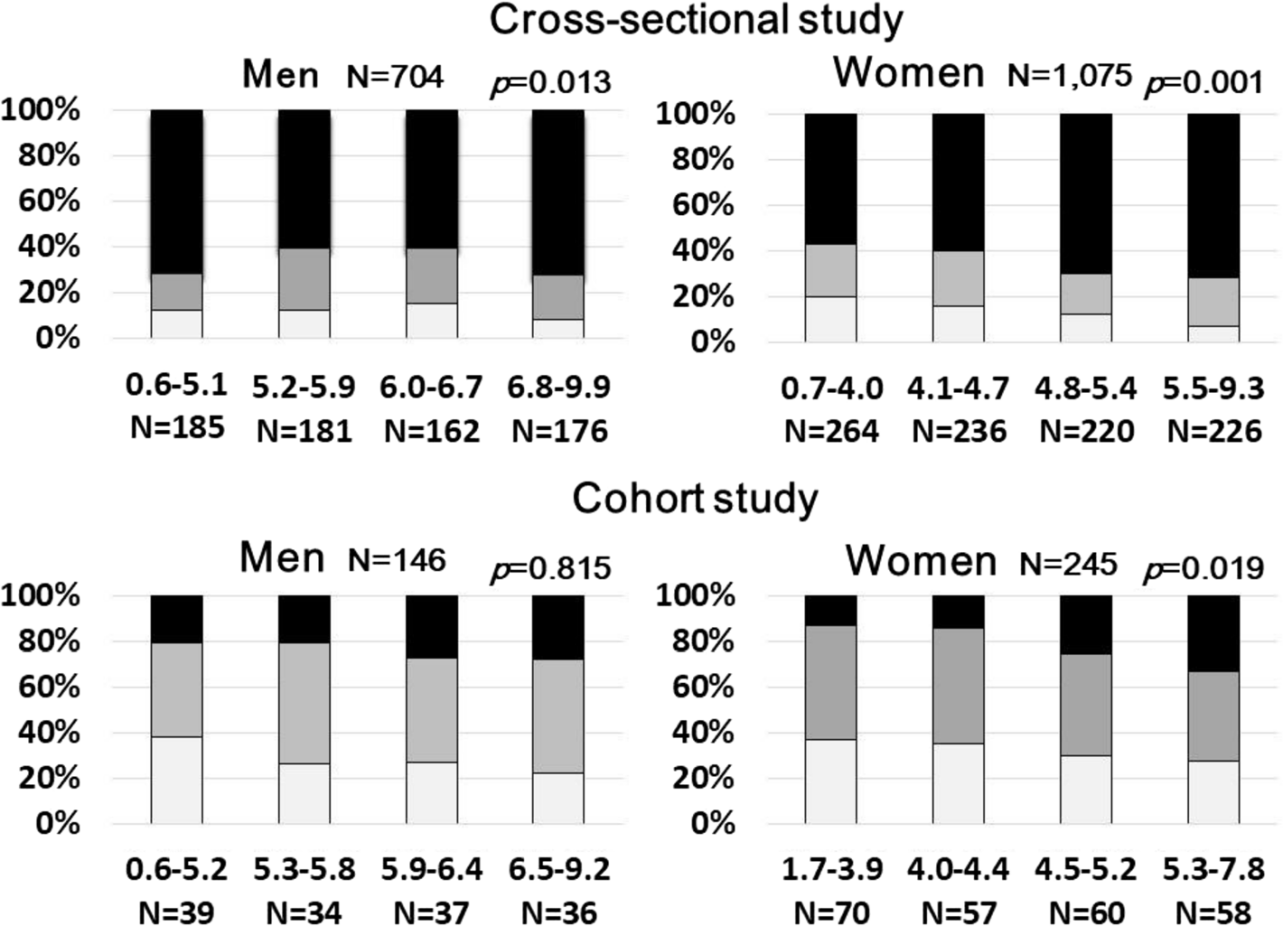
Prevalence and cumulative incidences of normotension (white box), prehypertension (gray box), and hypertension (black box) for each SUA level. Only in women, incidence of hypertension was found to increase with increasing concentrations of baseline SUA categories in both the cross-sectional (*p* = 0.001) and cohort studies (*p* = 0.019)

Figure 2 presents the prevalence and cumulative incidences of hypertension for each SUA level (men/women: SUA-1, < 5.2/< 4.1; SUA-2; 5.2–5.9/4.1–4.7; SUA-3, 6.0–6.7/4.8–5.4, SUA-4, ≥6.8/≥5.5 mg/dL). In the cross-sectional study, the respective number of subjects was 133 (71.9%), 109 (60.2%), 98 (60.5%), and 127 (72.2%) in men and 151 (57.2%), 141 (59.7%), 154 (70.0%), and 162 (71.7%) in women. In the cohort study (SUA-1, < 5.3/< 4.0; SUA-2; 5.3–5.8/4.0–4.4; SUA-3, 5.9–6.4/4.5–5.2, SUA-4, ≥6.5/≥5.3 mg/dL), the respective number of subjects was 8 (20.5%), 7 (20.6%), 10 (27.0%), and 10 (27.8%) in men and 9 (12.9%), 8 (14.0%), 15 (25.0%), and 19 (32.8%) in women. Only in women, incidence of hypertension was found to increase with increasing concentrations of baseline SUA categories in both the cross-sectional and cohort studies. However, in men, there was no inter-group difference regarding prevalence of hypertension.

### Odds ratios and 95% CI for hypertension of subjects according to baseline SUA categories by gender in the cross-sectional study

In the cross-sectional study, multiple logistic regressions were performed to evaluate the association between baseline SUA categories and hypertension (Table 3). Adjustments were made for the following variables: model 1 was unadjusted; model 2 was adjusted for age; and model 3, was further adjusted for smoking habit, alcohol consumption, and prevalence of CVD, LDL-C, TG, HDL-C, antilipidemic medication, HbA1c, antidiabetic medication, and eGFR. Only in women, in the unadjusted model, the odds ratios (ORs) [95% confidence interval (CI)] for hypertension comparing SUA-2, SUA-3, and SUA-4 to SUA-1 of SUA levels were 1.11 (0.78–1.59), 1.75 (1.20–2.55), and 1.89 (1.30–2.77), respectively. These associations were apparent even after further adjustments for age in model 2, but ORs were attenuated after all confounding factors in model 3. These associations were absent in men.

**Table 3:** Odds ratios and 95% CI for hypertension of subjects according to baseline serum uric acid in the cross-sectional study

Cross-sectional study	***N*** = 1650	Men ***N*** = 704			Women ***N*** = 946		
Baseline serum uric acid		Normotension/Hypertension			Normotension/Hypertension		
**Model 1**		**Unadjusted**			**Unadjusted**		
**Men/Women (mg/dL)**	**Total**	**N**	**odds ratio (95% CI)**	***p*-value**	**N**	**odds ratio (95% CI)**	***p*-value**
SUA-1 0.6–5.1/0.7–4.0	449	52/133	**1.0**	–	113/151	**1.0**	–
SUA-2 5.2–5.9/4.1–4.7	417	72/109	**0.59 (0.38–0.92)**	**0.019**	95/141	1.11 (0.78–1.59)	0.564
SUA-3 6.0–6.7/4.8–5.4	382	64/98	**0.60 (0.38–0.94)**	**0.025**	66/154	**1.75 (1.20–2.55)**	**0.004**
SUA-4 6.8–9.9/5.5–9.3	402	49/127	1.01 (0.64–1.61)	0.955	64/162	**1.89 (1.30–2.77)**	**0.001**
Continuous variable	1650	237/467	1.00 (0.87–1.15)	0.957	338/608	**1.27 (1.12–1.43)**	**< 0.001**
**Model 2**		**Age-adjusted**			**Age-adjusted**		
**Men/Women (mg/dL)**	**Total**	**N**	**odds ratio (95% CI)**	***p*-value**	**N**	**odds ratio (95% CI)**	***p*-value**
SUA-1 0.6–5.1/0.7–4.0	449	52/133	**1.0**	–	113/151	**1.0**	–
SUA-2 5.2–5.9/4.1–4.7	417	72/109	0.67 (0.43–1.05)	0.079	95/141	1.05 (0.73–1.53)	0.786
SUA-3 6.0–6.7/4.8–5.4	382	64/98	0.70 (0.44–1.10)	0.124	66/154	**1.84 (1.24–2.73)**	**0.003**
SUA-4 6.8–9.9/5.5–9.3	402	49/127	1.22 (0.76–1.95)	0.420	64/162	**1.83 (1.23–2.73)**	**0.003**
Continuous variable	1650	237/467	1.07 (0.92–1.23)	0.390	338/608	**1.27 (1.12–1.44)**	**< 0.001**
**Model 3**		**Multiple-adjusted**			**Multiple-adjusted**		
**Men/Women (mg/dL)**	**Total**	**N**	**odds ratio (95% CI)**	***p*-value**	**N**	**odds ratio (95% CI)**	***p*-value**
SUA-1 0.6–5.1/0.7–4.0	449	52/133	**1.0**	–	113/151	**1.0**	–
SUA-2 5.2–5.9/4.1–4.7	417	72/109	0.62 (0.38–1.00)	0.051	95/141	0.87 (0.58–1.30)	0.495
SUA-3 6.0–6.7/4.8–5.4	382	64/98	0.62 (0.37–1.03)	0.065	66/154	1.34 (0.87–2.07)	0.191
SUA-4 6.8–9.9/5.5–9.3	402	49/127	0.86 (0.50–1.49)	0.598	64/162	1.06 (0.66–1.69)	0.818
Continuous variable	1650	237/467	0.96 (0.81–1.13)	0.619	338/608	1.06 (0.91–1.23)	0.438

*CI* confidence interval. Multiple-adjusted for all confounding factors in Table 2. Bold values indicate significance (*p* < 0.05).

### Odds ratios and 95% CI for incident hypertension of subjects according to baseline SUA categories by gender in the cohort study

During a follow-up of 3.0 years, there were 35 (24.0%) hypertension cases in men and 51 (20.8%) in women (Table 4). Only in women, a significant association between increased SUA categories and incidence of hypertension was observed, and the ORs (95% CI) of incident hypertension of the SUA-3 and SUA-4 in model 1 were 2.26 (0.91–5.62) and 3.30 (1.36–8.03), respectively, and remained significant even when adjusted for age in model 2. Also, in the fully adjusted model, increased SUA remained an independent factor for incidence of hypertension, and the OR (95% CI) of SUA-4 was 3.84 (1.36–10.8). However, there was no significant association between SUA category and incidence of hypertension in men.

**Table 4:** Odds ratios and 95% CI for incident hypertension of subjects according to baseline serum uric acid in the cohort study

Cohort study	***N*** = 391	Men ***N*** = 146			Women ***N*** = 245		
Baseline serum uric acid		Normotension/Hypertension			Normotension/Hypertension		
**Model 1**		**Unadjusted**			**Unadjusted**		
**Men/Women (mg/dL)**	**Total**	**N**	**Odds ratio (95% CI)**	***p*-value**	**N**	**Odds ratio (95% CI)**	***p*-value**
SUA-1 0.6–5.2/1.7–3.9	109	31/8	1.0	–	61/9	**1.0**	–
SUA-2 5.3–5.8/4.0–4.4	91	27/7	1.01 (0.32–3.14)	0.994	49/8	1.11 (0.40–3.08)	0.846
SUA-3 5.9–6.4/4.5–5.2	97	27/10	1.44 (0.50–4.16)	0.505	45/15	2.26 (0.91–5.62)	0.080
SUA-4 6.5–9.2/5.3–7.8	94	26/10	1.49 (0.51–4.33)	0.463	39/19	**3.30 (1.36–8.03)**	**0.008**
Continuous variable	391	111/35	1.17 (0.83–1.64)	0.372	194/51	**1.54 (1.16–2.05)**	**0.003**
**Model 2**		**Age-adjusted**			**Age-adjusted**		
**Men/Women (mg/dL)**	**Total**	**N**	**Odds ratio (95% CI)**	***p*-value**	**N**	**Odds ratio (95% CI)**	***p*-value**
SUA-1 0.6–5.2/1.7–3.9	109	31/8	1.0	–	61/9	1.0	–
SUA-2 5.3–5.8/4.0–4.4	91	27/7	1.01 (0.32–3.18)	0.981	49/8	1.03 (0.37–2.89)	0.959
SUA-3 5.9–6.4/4.5–5.2	97	27/10	1.44 (0.49–4.19)	0.507	45/15	2.11 (0.84–5.29)	0.113
SUA-4 6.5–9.2/5.3–7.8	94	26/10	1.61 (0.55–4.73)	0.389	39/19	**2.97 (1.20–7.35)**	**0.018**
Continuous variable	391	111/35	1.19 (0.85–1.68)	0.313	194/51	**1.50 (1.12–2.00)**	**0.006**
**Model 3**		**Multiple-adjusted**			**Multiple-adjusted**		
**Men/Women (mg/dL)**	**Total**	**N**	**Odds ratio (95% CI)**	***p*-value**	**N**	**Odds ratio (95% CI)**	***p*-value**
SUA-1 0.6–5.2/1.7–3.9	109	31/8	1.0	–	61/9	1.0	–
SUA-2 5.3–5.8/4.0–4.4	91	27/7	0.87 (0.25–3.09)	0.833	49/8	1.25 (0.41–3.77)	0.692
SUA-3 5.9–6.4/4.5–5.2	97	27/10	1.59 (0.47–5.36)	0.456	45/15	2.23 (0.81–6.11)	0.119
SUA-4 6.5–9.2/5.3–7.8	94	26/10	1.33 (0.37–4.85)	0.662	39/19	**3.84 (1.36–10.8)**	**0.011**
Continuous variable	391	111/35	1.16 (0.78–1.74)	0.468	194/51	**1.59 (1.14–2.23)**	**0.007**

*CI* Confidence interval. Multiple-adjusted for all confounding factors in Table 2. Bold values indicate significance (*p* < 0.05)

## Discussions

In this cross-sectional and prospective 3-year follow-up cohort study, we set out to determine whether SUA within the normal range is a predictor of incident Hypertension. Baseline SUA within the normal range was significantly and independently associated with prevalence and incidence of hypertension, especially among women and can help clinicians to predict the progression of BP. To our knowledge, few studies have indicated that baseline SUA within the normal range could be an important potential factor for incidence of hypertension among community-dwelling elderly women.

In subjects with normal renal function, an increased SUA has been found to independently predict the development of hypertension [8, 21–24]. A recent systematic review found that elevated SUA levels were associated with incident hypertension [25, 26]. SUA has been shown to be a predictor of BP progression in most but not all studies [17]. A large cohort study among men who participated in the Health Professionals’ Follow-up Study showed that no independent association between SUA level and risk for incident hypertension was found among older men aged mean 61 years (range 47 to 81) [17]. Among 808 Korean participants during a mean follow-up of 3.3 years, 11.5% of men and 10.7% of women developed hypertension, and the association between SUA level and incident hypertension was positively significant among people aged < 55 years (relative risk 1.74 per 1.0 mg/dL of SUA; *p* = 0.002), but there was no significant association among people aged ≥ 55 years (*p* = 0.894) [14]. The possible influence of age on SUA-associated hypertension has been described by Sundstrom et al***.*** [8]; they noted a 13% increase in risk for each 1.0-mg/dl increase in SUA (mean age*.* 48.7 years), compared with a 20% increase for 1.0 mg/dl in the study by Taniguchi et al. [27] (mean age, 41 years) and a 23% increase for 1.0 mg/dl in that by Jossa et al. [28] (mean age, 36 years). In our study, the association between baseline SUA and hypertension was positively significant only among women aged ≥ 65 years.

Several previous studies have shown that SUA levels in the development of hypertension or kidney disease were significantly higher in women than in men [23, 29]. Additionally Lee et al. [30] have demonstrated that hyperuricemia increase the risk of hypertension in non-elderly patients (men < 60 years and women < 40 years). That is, it is conceivable that the effects of SUA in young persons may decrease over time and the higher incidence of hypertension induced by other causes with aging may reduce the strength of the relationship between SUA and hypertension [8, 30]. Thus, the effect of SUA might be eliminated because the association of SUA with hypertension largely reflects the predominance of metabolic risk factors such as increasing age, insulin resistance, dyslipidemia, renal dysfunction. Our study demonstrated that even SUA within the high normal range at baseline can be a risk factor in elderly people with normal blood pressure.

The mechanisms by which baseline SUA reflects the risk of hypertension are not fully understood. Uric acid (UA) is the final oxidation product of purine metabolism in humans and is excreted renally [31]. UA is catalyzed by the enzyme xanthine oxidase, which induces the production and destruction of free radicals, and also possesses dual pro-oxidant and antioxidant properties [32]. Potential mechanisms by which UA may cause hypertension have been previously published but include the ability of UA to induce intracellular and mitochondrial oxidative stress and decrease endothelium nitric oxide bioavailability as well as the intracellular renin-angiotensin system (RAS). Recent studies have reported that high UA may also reflect systemic inflammation and that cytokines such as C-reactive protein, interleukin-1, interleukin-6, and tumor necrotic factor α [33] are important predictors of incident CKD.

The strengths of this research are the fact that it is a long-term follow-up collection, the sample size, the adjustment for possible confounding factors, and the inclusion of sensitivity analyses. However, the authors acknowledge some limitations. First, our cohort study design could not eliminate potential causal relationships between baseline SUA and hypertension. The information on other factors (e.g., menopausal status or dietary habit) is insufficient to include in our analysis. Second, confounding factors and hypertension are based on a single assessment of blood and BP, so misclassification bias may occur. Third, we could not eliminate the possible effects of underlying diseases, medications for hypertension, dyslipidemia, diabetes, etc. on the present findings. Fourth, we have to assess the possibility that there were some participants with white-coat hypertension or masked hypertension. Therefore, generalization may be limited by demographics and referrals.

## Conclusions

This study showed that levels of baseline SUA in men contributed to incident hypertension, even after adjusting for baseline age, BMI, drinking status, smoking status, history of CVD, lipids, HbA1c, eGFR, and medication. The underlying mechanism behind this relationship is unknown. As such, lowering SUA levels by intervention (e.g., adopting a healthier lifestyle, medication) may prove to be a useful strategy for lowering hypertension burden. Further prospective population-based studies are needed to investigate SUA metabolism and eGFR by lifestyle interventions and medication.

## Data Availability

Not applicable.

## Abbreviations

CVD: Cardiovascular disease
SUA: Serum uric acid
RAS: Renin-angiotensin system
BMI: Body mass index
SBP: Systolic blood pressure
DBP: Diastolic blood pressure
JNC7: Joint National Committee 7
TG: Triglycerides
HDL-C: High-density lipoprotein cholesterol
LDL-C: Low-density lipoprotein cholesterol
HbA1c: Hemoglobin A1c
Cr: Creatinine
eGFR: Estimated glomerular filtration ratio
CKD-EPI: Chronic Kidney Disease Epidemiology Collaboration
SD: Standard deviation
ORs: Odds ratios
CI: Confidence interval
UA: Uric acid

## References

[1] Levey AS, Andreoli SP, DuBose T, Provenzano R, Collins AJ (2007). CKD: common, harmful, and treatable--world kidney day 2007. Am J Kidney Dis.

[2] Stamler J, Stamler R, Neaton JD (1993). Blood pressure, systolic and diastolic, and cardiovascular risks. US population data. Arch Intern Med.

[3] MacMahon S, Peto R, Cutler J, Collins R, Sorlie P, Neaton J (1990). Blood pressure, stroke, and coronary heart disease. Part 1, prolonged differences in blood pressure: prospective observational studies corrected for the regression dilution bias. Lancet (London, England).

[4] Lewington S, Clarke R, Qizilbash N, Peto R, Collins R (2002). Age-specific relevance of usual blood pressure to vascular mortality: a meta-analysis of individual data for one million adults in 61 prospective studies. Lancet (London, England).

[5] Grayson PC, Kim SY, LaValley M, Choi HK (2011). Hyperuricemia and incident hypertension: a systematic review and meta-analysis. Arthritis Care Res.

[6] Kang DH, Chen W (2011). Uric acid and chronic kidney disease: new understanding of an old problem. Semin Nephrol.

[7] Lyngdoh T, Marques-Vidal P, Paccaud F, Preisig M, Waeber G, Bochud M (2011). Elevated serum uric acid is associated with high circulating inflammatory cytokines in the population-based Colaus study. PLoS One.

[8] Sundstrom J, Sullivan L, D’Agostino RB, Levy D, Kannel WB, Vasan RS (2005). Relations of serum uric acid to longitudinal blood pressure tracking and hypertension incidence. Hypertension (Dallas, Tex: 1979).

[9] Mellen PB, Bleyer AJ, Erlinger TP, Evans GW, Nieto FJ, Wagenknecht LE (2006). Serum uric acid predicts incident hypertension in a biethnic cohort: the atherosclerosis risk in communities study. Hypertension (Dallas, Tex: 1979).

[10] Krishnan E, Kwoh CK, Schumacher HR, Kuller L (2007). Hyperuricemia and incidence of hypertension among men without metabolic syndrome. Hypertension (Dallas, Tex: 1979).

[11] Yokoi Y, Kondo T, Okumura N, Shimokata K, Osugi S, Maeda K (2016). Serum uric acid as a predictor of future hypertension: stratified analysis based on body mass index and age. Prev Med.

[12] Feig DI, Soletsky B, Johnson RJ (2008). Effect of allopurinol on blood pressure of adolescents with newly diagnosed essential hypertension: a randomized trial. Jama.

[13] Agarwal V, Hans N, Messerli FH (2013). Effect of allopurinol on blood pressure: a systematic review and meta-analysis. J Clin Hypertens (Greenwich, Conn).

[14] Lee SW, Kim HC, Nam C, Lee HY, Ahn SV, Oh YA (2019). Age-differential association between serum uric acid and incident hypertension. Hypertens Res.

[15] Kawamoto R, Ninomiya D, Senzaki K, Kumagi T (2018). Interaction between body mass index and serum uric acid in relation to blood pressure in community-dwelling Japanese men. Clin hypertens.

[16] Kawamoto R, Tabara Y, Kohara K, Kusunoki T, Abe M, Miki T (2014). Interaction between serum uric acid and triglycerides in relation to prehypertension in community-dwelling Japanese adults. Clin Exp Hypertens (New York, NY : 1993).

[17] Forman JP, Choi H, Curhan GC (2007). Plasma uric acid level and risk for incident hypertension among men. J Am Soc Nephrol.

[18] Wu AH, Gladden JD, Ahmed M, Ahmed A, Filippatos G (2016). Relation of serum uric acid to cardiovascular disease. Int J Cardiol.

[19] Kawamoto R, Ninomiya D, Kumagi T (2016). Handgrip strength is positively associated with mildly elevated serum bilirubin levels among community-dwelling adults. Tohoku J Exp Med.

[20] Horio M, Imai E, Yasuda Y, Watanabe T, Matsuo S (2010). Modification of the CKD epidemiology collaboration (CKD-EPI) equation for Japanese: accuracy and use for population estimates. Am J Kidney Dis.

[21] Perlstein TS, Gumieniak O, Williams GH, Sparrow D, Vokonas PS, Gaziano M (2006). Uric acid and the development of hypertension: the normative aging study. Hypertension (Dallas, Tex: 1979).

[22] Shankar A, Klein R, Klein BE, Nieto FJ (2006). The association between serum uric acid level and long-term incidence of hypertension: population-based cohort study. J Hum Hypertens.

[23] Zhang W, Sun K, Yang Y, Zhang H, Hu FB, Hui R (2009). Plasma uric acid and hypertension in a Chinese community: prospective study and metaanalysis. Clin Chem.

[24] Kuwabara M, Niwa K, Hisatome I, Nakagawa T, Roncal-Jimenez CA, Andres-Hernando A (2017). Asymptomatic Hyperuricemia without comorbidities predicts Cardiometabolic diseases: five-year Japanese cohort study. Hypertension (Dallas, Tex: 1979).

[25] Hwu CM, Lin KH (2010). Uric acid and the development of hypertension. Med Sci Monit.

[26] Feig DI (2012). The role of uric acid in the pathogenesis of hypertension in the young. J Clin Hypertens (Greenwich, Conn).

[27] Taniguchi Y, Hayashi T, Tsumura K, Endo G, Fujii S, Okada K (2001). Serum uric acid and the risk for hypertension and type 2 diabetes in Japanese men: the Osaka health survey. J Hypertens.

[28] Jossa F, Farinaro E, Panico S, Krogh V, Celentano E, Galasso R (1994). Serum uric acid and hypertension: the Olivetti heart study. J Hum Hypertens.

[29] Iseki K, Oshiro S, Tozawa M, Iseki C, Ikemiya Y, Takishita S (2001). Significance of hyperuricemia on the early detection of renal failure in a cohort of screened subjects. Hypertens Res.

[30] Lee JJ, Ahn J, Hwang J, Han SW, Lee KN, Kim JB (2015). Relationship between uric acid and blood pressure in different age groups. Clin Hypertens.

[31] Caliceti C, Calabria D, Roda A, Cicero AFG (2017). Fructose intake, serum uric acid, and Cardiometabolic disorders: a critical review. Nutrients.

[32] Berry CE, Hare JM (2004). Xanthine oxidoreductase and cardiovascular disease: molecular mechanisms and pathophysiological implications. J Physiol.

[33] Ruggiero C, Cherubini A, Ble A, Bos AJ, Maggio M, Dixit VD (2006). Uric acid and inflammatory markers. Eur Heart J.

